# Management of Complications of Portal Hypertension

**DOI:** 10.1155/2019/6919284

**Published:** 2019-06-04

**Authors:** Eduardo Garcia Vilela, Dominique Thabut, Marika Rudler, Paulo Lisboa Bittencourt

**Affiliations:** ^1^Clinic Hospital of Federal University of Minas Gerais, Brazil; ^2^Pitié-Salpêtrière Charles Foix Hospital, Paris, France; ^3^Portuguese Hospital of Salvador, Bahia, Brazil

Acute complications of portal hypertension are common conditions and have poor outcomes, despite the understanding regarding their pathogenesis. Portal hypertension develops when there is resistance to portal blood flow and it worsens as splanchnic blood flow increases. In this context, local release of vascular endothelial growth factor, nitric oxide, and other vasodilators that cause splanchnic arteriolar vasodilation occurs. The systemic repercussion of these changes leads to hypotension, vascular underfilling, and stimulation of endogenous vasoactive systems. As liver function decreases, there is a decrease in albumin synthesis and hyperaldosteronism, factors that are important in the development of ascites. In this issue, there are five articles that refer to the management of acute kidney injury, spontaneous bacterial peritonitis, and variceal bleeding.

Acute kidney injury (AKI) is directly associated with circulatory failure in cirrhosis, a phenomenon for which the main triggering factor is splanchnic arterial vasodilation secondary to portal hypertension. To compensate for the initial loss of effective arterial blood volume, counterregulatory neurohormonal systems are activated and produce substances such as renin, angiotensin, aldosterone, adrenaline, noradrenaline, and antidiuretic hormone. Thus, an attempt is made to restore intravascular volume through sodium and fluid retention and secretion of substances that would increase peripheral vascular resistance and cardiac output by increasing heart rate (the main determinant of cardiac output) that characterizes the hyperdynamic circulation in cirrhosis.

However, hyperdynamic circulation should not be considered an isolated phenomenon in the pathophysiology of kidney failure. In fact, AKI, as well as other forms of organ failure in cirrhosis, can occur even without the progression of circulatory failure because it results from the complex interaction between the innate immune system and bacterial components, also called pathogen-associated molecular patterns (PAMPs), translocated from the intestinal lumen and antigens from apoptotic cells, damage-associated molecular patterns (DAMPs). Once bound to pattern recognition receptors (PRRs), the PAMPs and DAMPs activate several intracellular and extracellular signalling cascades, producing proinflammatory responses that, when excessive or chronic, can cause tissue damage. Thus, AKI in cirrhosis should be interpreted as a complication resulting not only from arterial vasodilation but also from exacerbation of systemic inflammation. In this context, it is known that albumin molecule has the capacity to bind and decrease the biological effects of PAMPs, vasodilators released in systemic inflammation (NO and prostaglandins), and reactive oxygen species.

Spontaneous bacterial peritonitis (SBP) is defined as an ascitic fluid infection without an evident intra-abdominal surgically treatable source and it is considered a deleterious event in cirrhotic patients. It happens when bacteria within the gut lumen traverse the intestinal wall and colonize mesenteric lymph nodes. This phenomenon is associated with increase in intestinal permeability, promoted not only by portal hypertension but secondary to cytokine secretion, especially necrosis tumoral factor. Otherwise, the organism can move from the mesenteric lymphatics to the systemic circulation and then percolate through the liver and weep across Glisson's capsule to enter the ascitic fluid. Recently, an increase in drug-resistant bacterial infections has been described in cirrhotics patients. And previous hospitalization, nosocomial infection, and quinolone use for SBP prophylaxis have been associated with it. Besides resistance, a shift to gram-positive germs has also been shown, especially in SBP, which has been related to invasive procedures during hospitalization.

Acute variceal bleeding is a severe life-threatening complication of liver cirrhosis, accounting for 70% of all upper gastrointestinal bleeding in patients with portal hypertension. Although it is no longer the main cause of death in these patients, 6-week mortality reaches 15-20% per episode, mainly due to blood loss or multiple organ dysfunction. In all patients, splanchnic vasoconstrictors should be started as soon as variceal bleeding is suspected and continued for up to 5days to avoid early rebleeding. Endoscopy should be performed within 12 hours of admission, following hemodynamic resuscitation, to ascertain the cause of haemorrhage (up to 30% of cirrhotic patients bleed from nonvariceal causes) and to provide endoscopic therapy. Simultaneously to haemostatic-directed therapies, prevention of complications such as hepatic encephalopathy (HE) and bacterial infections should be put in place.

Despite therapy with vasoactive drugs plus endoscopic ligation, up to 10-15% of patients with acute variceal bleeding have persistent bleeding or early rebleeding. In such cases, transjugular intrahepatic portosystemic shunt (TIPS) should be considered as the rescue therapy of choice. When TIPS is not possible or in case of modest rebleeding, a second endoscopic therapy may be attempted while vasoactive drugs can also be optimized, hanging to terlipressin if not previously used. In refractory massive variceal bleeding, balloon tamponade or self-expanding metal stent may be used as temporary bridges to definitive therapy, such as TIPS or surgical shunt. In Asia, nonshunt surgery plus elective splenectomy is performed, too. Balloon-occluded retrograde transvenous obliteration (BRTO) can be considered in patients with refractory bleeding from gastric varices and in contraindication to TIPS, but it may increase portal hypertension following the procedure.

C. G. O. Gomes et al. evaluated the prognosis of patients with cirrhosis presented AKI and observed mortality of 28.6% in 30 days and 44.9% in three months despite use of albumin protocol. Advanced cirrhosis and nonresponders to volume expansion were associated with greater mortality.

J. D. C. Oliveira et al. analysed medical records related to culture-positive ascitic fluid from monobacterial SBP in cirrhotic patients. Multidrug resistant bacteria were isolated in 46.9% of samples and third-generation cephalosporin and quinolone resistant reached 38.9% and 25.7% of SBP cases. SBP due to multidrug resistant bacteria is a growing problem all across the world.

Y. Li et al. compared the outcomes of patients with liver cirrhosis and acute upper gastrointestinal bleeding who were admitted to hospital on regular hours and off-hours. After performing propensity score matching analysis, in-hospital mortality, 5-day rebleeding rate, length of hospital stay, and total payment were not different in two groups.

C. Bouzbib has issued TIPSS savage to treat refractory variceal bleeding. Despite its proven efficacy in control bleeding, this procedure is associated with very poor outcomes. The article discusses how to optimize management of those patients and some points were addressed such as which stents should be used, if concomitant embolization should be systematically considered, and why the prognosis with salvage TIPSS nowadays is as bad as earlier, despite the improvement of prophylaxis for variceal bleeding. Is there any alternative therapeutic in case of recurrent bleeding?

Finally, Y. Zhang et al. aimed to evaluate the safety of nonshunting methods to treat collateral veins in portal hypertension when other less invasive methods are not indicated or unavailable. They also do elective splenectomy to treat hipersplenism and justify it since hepatitis B virus is the most cause of portal hypertension and liver cirrhosis.

